# Liquid Moisture Transport in Cotton Woven Fabrics with Different Weft Yarns

**DOI:** 10.3390/ma15186489

**Published:** 2022-09-19

**Authors:** Małgorzata Matusiak, Dominika Kamińska

**Affiliations:** 1 Lodz University of Technology, Faculty of Material Technologies and Textile Design, Institute of Architecture of Textiles, 90-924 Lodz, Poland; 2 Institute of Security Technologies MORATEX, 90-505 Lodz, Poland

**Keywords:** cotton 1, woven fabrics 2, liquid transport 3

## Abstract

Moisture transport in fabrics influences the thermal comfort of clothing due to drainage of sweat secreted by the human body. The moisture transport through textile materials takes place in two ways: water-vapor transport and liquid moisture transport. Both ways are equally important. In the present work, liquid moisture transport in cotton woven fabrics with different weft yarns was investigated. Measurement was done using the Moisture Management Tester MMT M290. The obtained results confirmed that the linear density of weft yarn significantly influenced the values of all parameters characterizing liquid moisture transport in the investigated fabrics. The best performance in liquid moisture transport was achieved by weft yarn of linear density 30 tex. For this fabric variant, the maximum wetted radius for both surfaces was the biggest: 25 mm for the inner and 26.6 mm for the outer surface of the fabric. This means that the fabric spread the liquid on the biggest area compared to the other variants being investigated to facilitate an evaporation of liquid sweat. The fabric variant with 30 tex weft yarn showed the highest spreading speed: 5.83 mm/s for both sides, and the shortest wetting time: 2.83 s for the inner and 3.00 s for the outer side of the fabric. The higher the linear density of weft yarn, the worse the ability of cotton woven fabrics to ensure liquid moisture transport.

## 1. Introduction

Moisture transport is one of the most important comfort-related properties of fabrics and clothing. It influences the transport and evaporation of sweat produced by the human body. Among other things, clothing plays a role in thermal regulation [1]. It is a barrier between the human body and surroundings. It influences both the heat flow between the human body and environment, as well as the moisture and air exchange between underclothing and environment. Clothing protects the human body against both excessive heat loss and overheating. The moisture transport in fabrics influences thermal comfort due to drainage of sweat secreted by the human body [2]. Sweat is produced permanently by the human body, although the sweating intensity can be different. According to the literature, it is from almost zero to 5 L per hour. For instance, the whole body’s overall sweating rate due to exercise has been determined at a level of 1.13 L/h [3]. The intensity of sweating depends on different factors: climatic conditions, intensity of activity, and innate features of the human organism.

Sweating is the most important active mechanism of heat loss. Evaporation of 1 L of sweat from the surface of the skin consumes more than 2400 kJ (573 kcal). The maximum sweat secretion in an acclimatized person is 1.5 L per hour. During the acclimatization period to particularly hot climatic conditions, perspiration can be as high as 4 L per hour with heat loss of 10,000 kJ (2399 kcal). Due to this fact, moisture transport is one of the important elements of heat balance of the human organism. However, sweating causes heat loss only in cases when water evaporates from the skin’s surface.

Moisture transport through clothing takes place in two ways [4]:

- Water-vapor transport;
- Liquid moisture transport.

It has been found that the steady state vapor transport and moisture liquid transport through textile fabrics are two independent behaviors. It is also stated that there is no significant correlation between the steady state vapor resistance and the overall one-way transport capacity [2]. Water-vapor transport concerns moisture in the form of gas. The water-vapor permeability of textile materials applied in clothing supports the moisture transfer from the skin of the human body through the textile layers into the environment [5]. It is connected with the diffusion of the water-vapor molecules through the pores in fabrics. This property is important, especially while higher levels of activity and/or climatic conditions cause intensive sweating. In such a situation, the sweat must be rapidly managed by clothing [1]. The water-vapor permeability of fabrics depends mostly on the structure of fabrics, especially their porosity. Water vapor is transmitted through the pores existing in the clothing materials, both between the fibers in yarns creating the fabrics and between the yarns in woven and knitted fabrics. Here, the crucial role is played by the open pores in fabrics. The water-vapor resistance of textile materials can be determined using the sweating guarded hot plate method [6], also called the “skin model”, or the Permetest by Sensora (Czech Republic) [7,8].

Water-vapor permeability characterizes fabrics from the aspect of transport of moisture in the form of vapor. It is insufficient to characterize in a complex manner textile materials from the point of view of their moisture transport from the human skin to the environment. In order to fully assess textile materials from the point of view of physiological comfort, it is necessary to measure the materials from both aspects: the transfer of moisture in the form of vapor and in the form of liquid.

Sweat in a liquid form occurs at high sweating rates and it wets clothing that is in contact with the human skin. The liquid moisture flow through the textile materials is controlled by two processes: wetting and wicking. The term “wetting” is usually used for description of the displacement of a solid-air interface with a solid–liquid interface [9]. It is an initial process, involved in fluid spreading on the fabric surface. This process is controlled by the surface energies of the involved solid and liquid [10]. Wettability is the potential of a surface to interact with liquids with specified characteristics [11]. According to Harnett and Mehta [12], wettability is the initial behavior of the fabric, yarn or fiber when brought into contact with a liquid. It also describes the interaction between the liquid and the substrate prior to the wicking process.

Wicking is a spontaneous flow of liquid in porous materials, such as fabrics. The flow is driven by capillary forces. Wicking means movement of the liquid into the capillary spaces of the fabric: between the fibers in yarns and between the yarns in fabrics [10,13]. Wicking can only take place when the liquid wets fibers creating the textile material due to the capillary spaces existing between them. The resulting capillary forces drive the liquid into the capillary spaces [11,13]. Thinner gaps between the individual fibers cause increase of the capillary forces. Thus, finer fibers will create smaller gaps in the fabric structure, and in consequence better moisture transport.

The wicking in textile materials is a very complex phenomenon. Generally, we should distinguish vertical and horizontal wicking. In vertical wicking tests, the fabric sample is placed vertically and the bottom of the specimen comes into contact with water. Then, the wicking distance by specified time intervals is recorded. The higher the wicking distance at the same interval, the better the fabric is in wicking [14].

The height of a liquid column in a capillary is given by Jurin’s law [15]:

(1) $$h=\frac{2\gamma \;cos\theta }{\rho gr}$$

where:

*h*—height of liquid in capillary;
γ—the liquid-air surface tension (force/unit length);
*θ*—the contact angle;
*ρ*—the density of liquid (specific mass/volume);
*g*—the local acceleration due to gravity.

The vertical wicking test is commonly used for an assessment of the liquid transport through textile materials. It makes it possible to compare different fabrics from the aspect of wicking. However, this test method does not reflect the real wicking phenomenon while clothing is used. In such a situation, liquid sweat occurs on the human skin surface. The orientation of the human skin surface is different depending on the place on the human body. Sometimes, it is a vertical orientation when drops of liquid occur on the vertically oriented surface. In other cases, the orientation of the skin surface is horizontal and/or inclined at a certain angle to the vertical. The surface of clothing covering the human body usually has the same orientation as the orientation of the human skin surface covered by the clothing.

In the horizontal wicking test [16], a specific amount of water is used from a specific height. The time of the water spreading out from the center to the edge of the circle (100 mm in diameter) is recorded. In this method, the shorter the time is, the better the wicking. In the horizontal wicking test, the spreading of the liquid is assessed only on the upper surface of the fabric. From the point of view of the physiological comfort of clothing usage, the spreading of the liquid on both surfaces of fabric—the upper and the bottom—is equally important. The horizontal wicking test does not provide any information concerning the spreading of the liquid on the bottom surface. Taking this into account, we can state that the results from both wicking tests—the vertical and the horizontal—are insufficient when assessing fabrics designed for clothing, especially for clothing worn in direct contact with the human skin.

The moisture transmission in the liquid and the vapor form are equally important from the point of view of the thermo-physiological comfort of clothing users [3]. Wearing garments that transport moisture and evaporate it quickly significantly enhances the human body’s ability to cool itself [3].

The water-vapor permeability of fabrics depends mostly on the fabric structure, especially its porosity [17]. The fabric structure also influences the liquid moisture transport through the fabrics because the structure of fabrics affects the capillary spaces in them [18]. The ability of fabrics to transport liquid moisture can be changed and/or shaped by appropriate finishing [19,20]. Su Kyoung An et al. investigated several patented knitted fabrics designed for athletic apparel. The investigated fabrics had been finished using moisture management finishing technologies. The function of the investigated fabrics was to keep the human skin dry and comfortable. However, the performed investigations showed that the effectiveness of moisture management finishing technology in heat and sweat transfer was insufficient [19]. Udaya Krithika et al. successfully applied amino silicone polyether copolymers and hydrophilic polymers as moisture management finishing agents to improve moisture management properties of cotton, cotton/polyester, and nylon woven fabrics. They observed that among wicking, water absorbency, wetting, and moisture vapor transfer tests, the effect in fabrics with wetting agents was significantly better than that of fabrics treated without wetting agents, especially for the fabrics made of cotton and microdenier polyester [20].

The raw material of fabrics is also an important factor influencing the liquid moisture transport in textile materials and clothing. An especially crucial role is played by the hydrophilicity of fibrous material. It influences the wetting ability of fabrics. Traditionally, materials made of natural plant fibers such as cotton are valued for their excellent intrinsic hygienic properties [21,22]. This is because these fibers are hydrophilic and in consequence they absorb moisture. Cotton fibers are built of pure cellulose, a naturally occurring polymer. In cellulose molecules there are negatively charged OH groups on the outer edge. These groups attract the water molecules and make cellulose and cotton absorb water well. These hydroxyl groups of cellulose bond water inside the cotton fibers [23].

Due to its hydrophilicity, cotton is often used in underwear and summer garments, especially those worn in direct contact with the skin. In contrast to synthetic fibers, the cotton fibers absorb water which remains inside the fabric. It is considered as a disadvantage of cotton products from the viewpoint of thermo-physiological comfort [24]. The absorption of water by cotton fibers means the transport of liquid by the capillary forces in the cotton fabrics is limited. Generally, the transport of liquid moisture through cotton fabrics is the result of two phenomena: absorption and capillarity. By an appropriate engineering of cotton fabrics it is possible to shape the phenomenon of the liquid moisture transport in cotton textile materials and clothing. The structure of woven fabrics can be changed by the selection of the linear density of the warp and weft yarns, the density of yarns (the number of picks and ends) and the weave. All of these factors affect a majority of the properties of woven fabrics, including the transport of moisture in both water vapor and liquid forms. Investigations [25] have confirmed that the weave significantly influences the transport of liquid moisture through cotton woven fabrics. Six different weaves—plain, twill 3/1S, twill 2/2 S, rep 1/1 (010), rep 2/2(2), and hopsack 2/2(020)—were used in the investigated cotton woven fabrics. The obtained results confirmed that the fabric with the hopsack 2/2 (020) weave was characterized by the best performance in transport of liquid moisture. The worst performance was found in the plain woven fabric. The influence of other structural parameters of the woven fabrics, such as the density of weft and warp as well as the thickness of threads, has not been investigated till now. As mentioned above, the basic structural parameters of woven fabrics have a significant influence on a majority of the comfort-related and utility properties of the fabrics. Numerous investigations confirmed that the density of yarns and their thickness influence the derivative fabric structure parameters, such as the surface cover and the filling, and in consequence their porosity, air permeability, water-vapor resistance, and many others [25]. The influence of the yarn thickness and density on the liquid moisture transport through woven fabrics has not been investigated till now. A majority of the investigations of this aspect concern knitted fabrics [26,27,28], which is a significant problem related to the physical comfort of using clothing made of woven fabrics. The warp and weft thickness and density affect the density of the fabric structure, and thus the size, size distribution, and spatial orientation of the spaces between the fibers and between the yarns in the woven fabrics.

Taking this into account, the aim of the presented work was to investigate the influence of the linear density of weft yarn on the liquid transport properties of cotton woven fabrics. In further work, the influence of the density of weft and warp yarns on the liquid moisture transport in woven fabrics will be investigated too. Together with the results of the work aimed at assessing the influence of the weave on liquid moisture transport [25], new knowledge will be created by describing and explaining in detail the influence of woven fabrics’ structure on the liquid moisture transported through them.

## 2. Materials and Methods

Woven fabrics of twill 3/1 S weave were the subjects of the investigations. In order to assess the influence of the weft linear density on liquid moisture transport, fabrics have been designed and manufactured in such a way to eliminate other factors influencing moisture transport. This means that all the investigated variants were made of the same raw material—cotton. This allowed us to eliminate the influence of fibers’ properties on the liquid moisture transport through the investigated fabrics. The fabrics were manufactured from the same warp yarn—50 tex cotton (CO) open end (OE) yarn. All fabric variants were manufactured on the same loom. The same nominal warp and weft densities were used on the loom during manufacturing:

- Nominal warp density—320 threads/dm;
- Nominal weft density—110 threads /dm.

In order to assess the influence of the weft yarn on the liquid moisture transport through the fabric, 5 kinds of cotton OE yarns were used as a weft: 30 tex, 40 tex, 50 tex, 60 tex, and 100 tex. These are typical of the cotton OE yarns applied in the cotton industry.

All fabric variants were dyed and finished in the same way—with starch finishing, typical of cotton fabrics. The conditions of finishing were identical for all fabric variants. However, due to the different weft yarns applied in the investigated fabrics, the relaxation of fabrics after the weaving and finishing processes ran in slightly different ways for each fabric variant. This caused the final (real) values of the warp and weft density to be different for particular variants than that adjusted on the loom. The basic structural parameters of the woven fabrics investigated are presented in Table 1. The measurements of the structural parameters of the fabrics were performed using appropriate standardized procedures:

- Warp and weft density according to the ISO 7211-2:1984 standard [29];
- Mass per square meter according to the ISO 3801:1977 standard [30];
- Take-up of warp and weft according to the PN-88/P-04636 standard [31];
- Thickness according to the ISO 5084:1996 standard [32].

An increase of the linear density of the weft yarn caused an increase of the take-up of the warp yarn as well as the mass per square meter and thickness of fabrics. The take-up of the weft yarn decreased with the increase of weft linear density.

**Figure 1:**
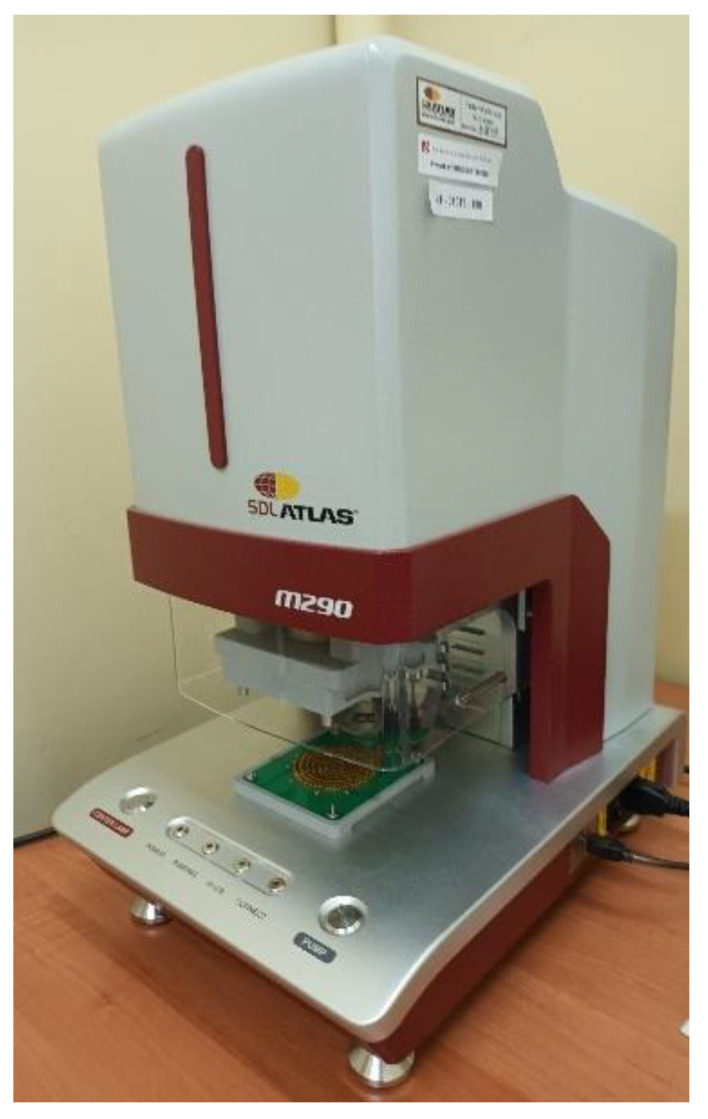
The Moisture Management Tester M 290 by SDL Atlas.

The manufactured fabric variants have been tested in the range of the liquid moisture transport through them. The measurement was performed by means of the Moisture Management Tester (MMT) M290 (Figure 1) by the SDL Atlas Rock Hill, SC, USA. This is an instrument designed to measure the dynamic liquid transport properties of textile materials in three aspects [24,25,33,34,35,36,37]:

- The absorption rate—moisture absorbing time for inner and outer surfaces of the fabric;
- The one-way transport capability—one-way transfer of liquid moisture from the inner surface to outer surface of fabric;
- The spreading/drying rate—speed of liquid moisture spreading on the inner and outer surfaces of fabric.

In the presented method, the inner surface of fabric means the surface which adheres to the user’s skin while wearing the clothing.

The MMT device was used with a PC and the MMT290 software. The measurement was performed according to the testing procedure described in the MMT manual [30] based on the AATCC Method 195 standard [37]. According to the applied procedure, the measurement was performed for samples cut into 80 mm × 80 mm squares. Normally, the procedure consists of 5 repetitions [33] for each investigated fabric variant. However, previous investigations [25] showed a large variation of results from the MMT for the woven fabrics. Due to this fact, it was decided to perform 10 repetitions for each fabric variant while measuring a predefined amount of test solution imitating human sweat introduced onto the upper inner side (skin side) of the fabric. Next, the testing solution was transferred onto the material in three directions [33]:

- Spreading outward on the upper surface of the measured specimen;
- Flowing from the upper surface to the bottom surface through the fabric;
- Spreading outward on the bottom surface of the measured specimen.

Measurements were performed in standard climatic conditions: 65 ± 5% RH and ambient temperature 20 ± 2 °C. The following parameters were determined as the results of measurement using the MMT:

- The wetting time for top (WTT) and bottom (WTB) surface in s;
- The absorption rate for top (TAR) and bottom (BAR) surface in %/s;
- The maximum wetted radius for top (MWR_top_) and bottom (MWR_bottom_) surface in mm;
- The spreading speed for top (TSS) and bottom (BSS) surface in mm/s;
- The accumulative one-way transport index R, -;
- The overall moisture management capability (OMMC), -.

In order to assess the influence of the weft yarn thickness on the liquid moisture transport through the fabrics, statistical analysis was performed using the one-way ANOVA available in the TIBC^®^ STATISTICA™ version 13.3 software. The analysis of variance (ANOVA) was applied to assess the significance of differences between means. In the applied software, the ANOVA compares two variances: the variance due to the between-groups variability (called mean square effect, or MS_effect_) and the variance due to the within-group variability (called mean square error, or MS_error_). The comparison was done using the F test. The F-test checks whether the ratio of the two variances is significantly bigger than 1. These latter variance components were then tested for statistical significance, at the significance level 0.05 [38]. In the applied software, the interpretation of the results was the following:

- When *p* ≤ 0.05 there is statistically significant difference between within-group and between-groups variability;
- When *p* > 0.05 the difference between within-group and between-groups variability is statistically insignificant.

In the performed statistical analysis, the linear density of the weft yarn was applied as a main factor (the independent variable). Particular parameters determined by means of the MMT have been taken as the dependent variables.

In order to assess the differences between particular means, the Tukey’s test was applied. It is one of the post hoc tests allowing a single-step multiple comparison of means. The Tukey’s test is used for finding the means that are significantly different from each other at the applied (0.05) significance level. [38]. Interpretation of the results is similar to the interpretation of the ANOVA results; i.e., when *p* ≤ 0.05 it means that the difference between compared means is statistically significant.

## 3. Results and Discussion

The results of measurements of the cotton woven fabrics by means of the MMT are presented in Table 2 and Table 3. Detailed results are presented in the Supplementary Materials (Tables S1–S5).

As we can see, the values of particular parameters characterizing the liquid moisture transport through the investigated fabrics were different although the fabrics were made of the same raw material (cotton) and manufactured with the same weave and the same thread densities adjusted on the loom. The application of weft yarns of different linear densities caused the differences in the fabric structure, which in consequence caused the changes in the liquid moisture transport.

In order to assess the significance of influence of the weft yarn linear density on the liquid moisture transport properties, statistical analysis was performed. The results of the statistical analysis using the one-way ANOVA are presented in Table 4.

The statistical analysis confirmed that the linear density of the weft yarn influenced all parameters characterizing the liquid moisture transport in the cotton woven fabrics. In all cases, the influence was statistically significant at the significance level 0.05.

**Figure 2:**
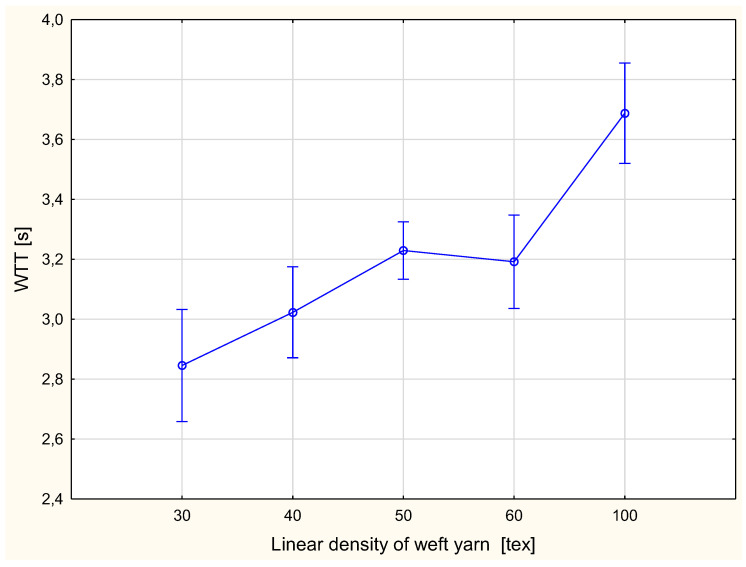
Wetting time for the top fabric surface vs. linear density of the weft yarn.

**Figure 3:**
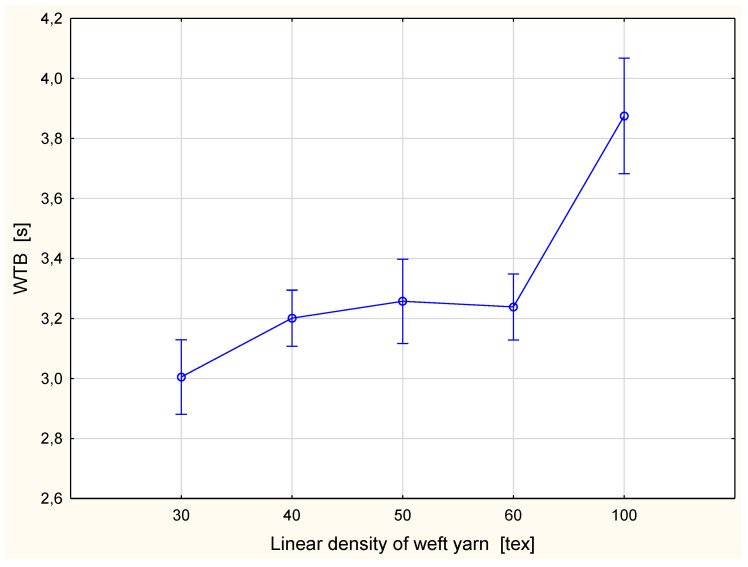
Wetting time for the bottom fabric surface vs. linear density of the weft yarn.

**Figure 6:**
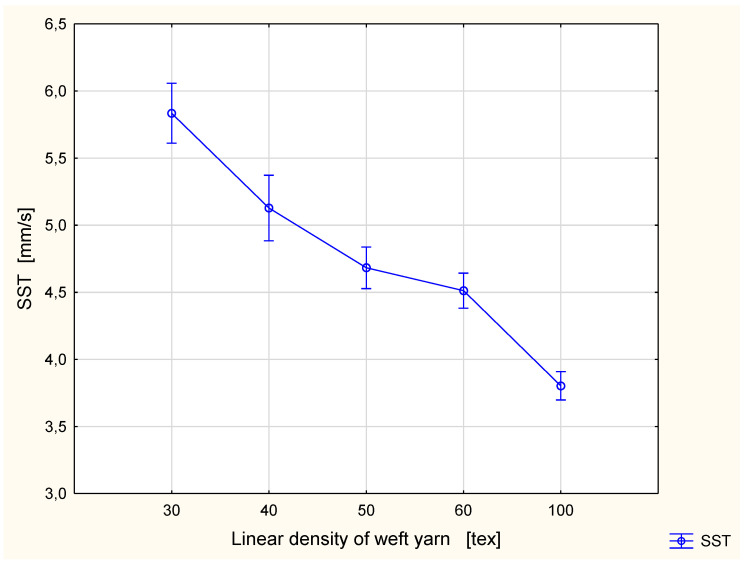
Spreading speed for the top fabric surface vs. linear density of the weft yarn.

**Figure 7:**
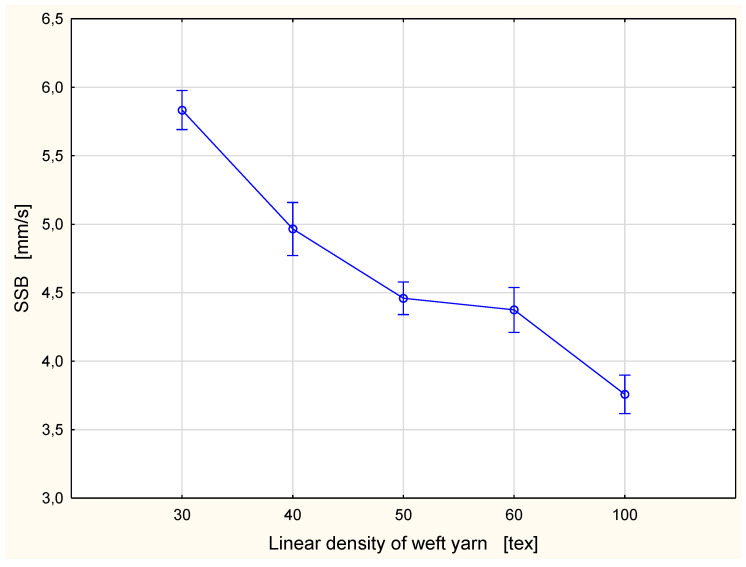
Spreading speed for the bottom fabric surface vs. linear density of the weft yarn.

**Figure 8:**
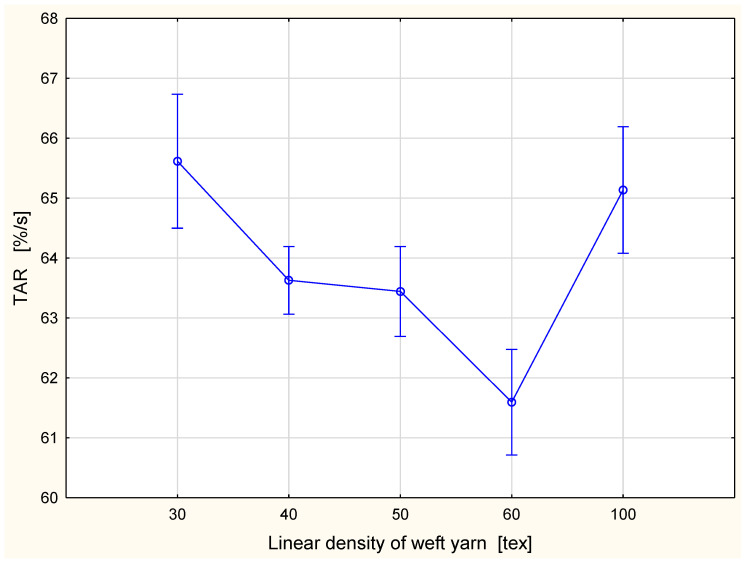
Absorption rate for the top fabric surface vs. linear density of the weft yarn.

**Figure 9:**
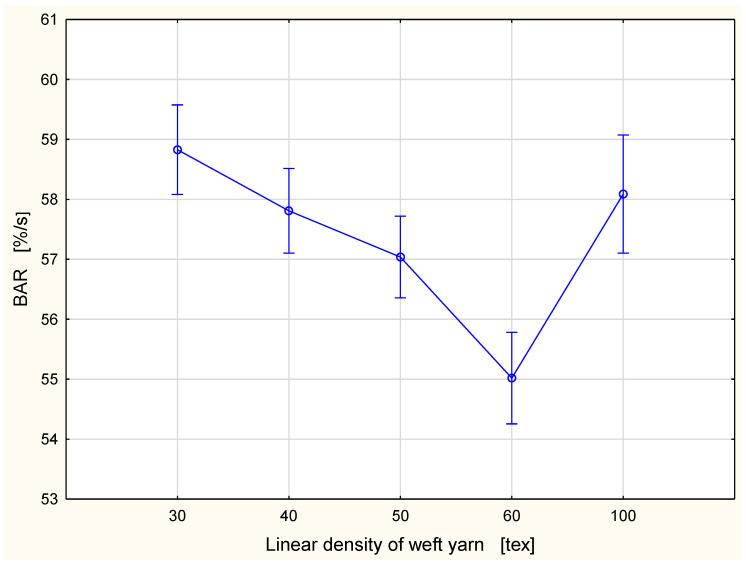
Absorption rate for the bottom fabric surface vs. linear density of the weft yarn.

The detailed results are presented in Figure 2, Figure 3, Figure 6, Figure 7, Figure 8, Figure 9, Figure 10, Figure 11, Figure 14 and Figure 15. The graphs show the mean values of particular parameters and the confidence intervals at the confidence level 0.95%. Generally, it can be seen that the confidence intervals are broad. A large scattering of MMT results has been found in previous studies [25], as we mentioned earlier. Due to this fact, 10 repetitions of measurement were performed for each fabric variant.

On the basis of the presented results, it is difficult to state whether the relatively large scatter of the results was caused by an uneven structure of the tested fabrics or the phenomenon of the liquid moisture spread on the fabrics’ surface. There are no data on this topic in the literature. The test solution was dosed pointwise onto the tested fabric surface. Sometimes the drops of the testing solution fell onto the pores between the threads in fabric and sometimes on the warp or weft thread, depending on the fabric weave and placement of the measured specimen on the device. In the investigated fabric variants, the warp and weft yarns were different. All yarns were made of cotton. However, due to different linear densities of the warp and weft, the yarn twist was also different. This influences the packing density of the yarns, and in the same way the capillarity of yarns. All mentioned factors should be considered as the reasons for the rather big variation of the results from the MMT.

Figure 2 and Figure 3 show the wetting time for the top (Figure 2) and bottom (Figure 3) surfaces of the fabrics.

The WTT and WTB are the time periods in which the top and bottom surfaces of the fabric started to become wet after the test commenced. They are defined as the time in second (s) [33]. The longer the wetting time was, the worse the performance of the fabric from the aspect of wettability. The shortest wetting time was for the fabric variant with the 30 tex weft yarn. For the top surface, the value of the WTT was 2.84 s, and for the bottom surface the WTB was 3.00 s. The longest wetting time for both sides occurred for the fabric variant with the 100 tex weft yarn: the WTT was 3.69 s, and the WTB 3.88 s. It was also found that the wetting time for fabric variants with the weft yarns 50 tex and 60 tex was at the same level. For the fabric variant with the 50 tex weft yarn, the WTT value was 3.23 s and the WTB was 3.26 s, whereas for the variant with the 60 tex weft yarn the WTT was 3.19 s and the WTB 3.24 s. For both surfaces, inner and outer, the tendencies were the same. The wetting time increased with an increase of the linear density of weft yarn. This means that when using coarse weft yarn, the wettability of fabrics becomes worse. This results from the fact that the structure of the fabric changes due to the change of the linear density of the weft yarn, although other structural parameters are unchanged. Using thicker weft yarn while the density of weft yarn is the same causes the pores between the weft yarns to decrease.

**Figure 4:**
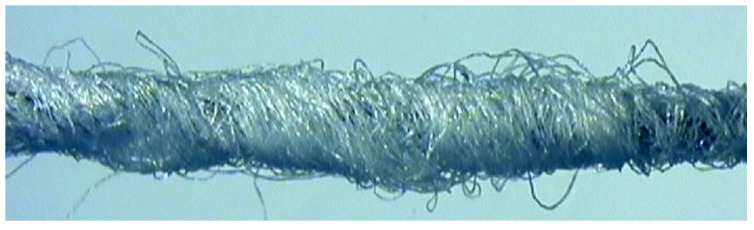
Exemplary microscopic picture of the OE cotton yarn.

**Figure 5:**
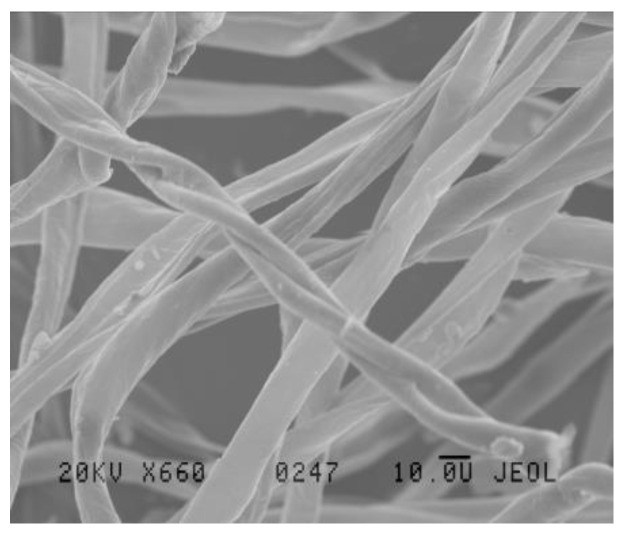
Exemplary microscopic picture of cotton fibers.

It should be mentioned here that it was difficult to determine the size of pores and the pore size distribution in the investigated fabrics. The fabrics were characterized by a compact structure without any open pores. Due to this fact, the transport of the liquid moisture driven by the capillary forces occurred in the micropores between the fibers and between the yarns. It was difficult to assess the size of a cross-section of pores because they were very small and irregular. Additionally, their spatial arrangement was diversified and unpredictable. The fibers in cotton yarns made by open end rotor spinning technology are oriented randomly and differently in the core and covering phase of the yarn (Figure 4). Determination of size of the spaces between the fibers in yarns and the size distribution requires advanced measuring techniques utilizing optical and computational technologies. It is also commonly known that cotton fibers do not have cylindrical shape although such shape is often used for modeling the structure of yarns and fabrics [39]. In reality, cotton fibers have a ribbon-like twisted shape (Figure 5). This additionally makes it difficult or even impossible to determine the size of the pores between the fibers in the cotton yarn.

Additionally, the yarns in the woven fabrics were oriented in different ways depending on their place on the fabric surface. Some yarn sections were arranged horizontally, with fragments oriented vertically, and in some parts at an angle to the horizontal in the places where the warp and weft were intertwined.

Szosland [40,41] analyzed the structure of the inter-thread channels in woven fabrics. In presented models he assumed that in the fabric structure there were open pores and the yarns were cylindrical solid objects with smooth walls. The blocks reflecting the shape of the inter-thread space in the fabric models were very complicated and irregular although the author made a lot of simplifications. Additionally, in the case of fabrics made of cotton yarns, the shape of yarns was not cylindrical: the yarns were not solid objects but in the form of a stream of fibers of numbers that were different in cross-section and different in packing density [42]. The walls of cotton yarns are not smooth (Figure 4). In reality, determination of the pore size and the pore size distribution in cotton woven fabrics without open pores is very difficult and may be subject to large error. In order to analyze the structure of cotton woven fabrics, the surface cover factor and the volume filling can be determined, but neither parameter reflects the shape and size of capillaries inside the fabric.

All factors discussed above influence the capillarity process, but simultaneously, coarse weft yarn means a larger amount of fibrous material in the fabric structure. In the case of the investigated fabric variants, all were made of cotton, which is hydrophilic and absorbs water. Due to this fact, a larger amount of cotton fibers in the fabric structure causes higher absorption of water. Both factors—the size of pores between yarns and larger amount of fibrous material—interact in the opposite way in the transport of liquid in the fabric. The final results should be considered as the result of the impact of individual factors. It also should be mentioned here that in coarse yarn there is a larger number of fibers in yarn cross-section than in thinner yarn made of the same fibers. This also influences the conditions of the liquid transport in the fabric due to the larger number of pores between fibers.

These results show that for all fabric variants, the wetting time for the inner (upper) surface is shorter than for the outer (bottom) surface. This is understandable as it also takes some time for the fluid to pass from the top to the bottom surface.

The results of the wetting time tests were in agreement with the results of the spreading speed tests for both surfaces (Figure 6 and Figure 7). The spreading speed is defined as the accumulative spreading speed from the center (the point of dosing the testing solution) to the maximum wetted radius [33]. The higher the spreading speed is, the better the spreading of the liquid on the fabric surface, and in consequence the better are conditions for liquid sweat evaporation. In both cases, the SST and the SSB, the lowest spreading speed was found for the fabric with the 100 tex weft yarn. For this fabric variant, the value of the SST was 3.80 mm/s, and the SSB 3.76 mm/s. The highest spreading speed was found for the fabric variant with the 30 tex weft yarn. The values of the spreading speed for this variant were the same for both surfaces—5.83 mm/s. For both fabric surfaces, the spreading speed decreased with the increase of the linear density of the weft yarn applied in the investigated fabric. This means that using weft yarn of higher linear density when other structural parameters (warp linear density, warp density, weft density and weave) are the same causes a worsening of fabric performance from the aspect of liquid moisture transport. The liquid is spread slower, and simultaneously it is evaporated slower too.

A different tendency was observed in the case of the absorption rate. The TAR (Figure 8) and the BAR (Figure 9) are defined as the average speed of the liquid moisture absorption for the top and bottom surfaces of the specimen during the initial change of water content during a test [34]. They are expressed as a percentage per second. The parameters are determined from the “water content vs. time” graph available in the MMT M290 software as the slopes between where the specimen begins to wet and the maximum point on the graph [33]. The highest value of the absorption rate occurred for the fabric variant with the 30 tex weft yarn: the TAR was 65.62%/s and the BAR 58.83%/s. The lowest absorption rate was found for fabric with the 60 tex weft yarn: the TAR was 61.59%/s and the BAR 55.02%/s. In both cases, the TAR and the BAR values of parameters decreased with the increase of the linear density of the weft yarn. Such a situation was observed for fabrics with weft yarns in the range 30–60 tex. Next, the value of both parameters increased significantly for the fabric variant with 100 tex weft yarn and had the following values: the TAR was 65.13%/s and the BAR 58.09%/s. The values of the absorption rate for the fabric variant with the 100 tex weft yarn were slightly lower than those for the fabric variant with the 30 tex weft yarn. It is difficult to explain this phenomenon. In our opinion, it resulted from an interaction between the linear density of the weft yarn and other structural parameters of fabrics. The fabric variant with the 100 tex weft yarn contained the largest amount of fibrous material—cotton. Cotton fibers are hydrophilic and they absorb liquid (water) well. At the same time, a higher absorption of liquid is in opposition to the transport of the liquid due to capillarity. On the other hand, for fabrics with weft yarns of lower linear density, liquid moisture may be transferred down by the interstitial spaces in the fabric due to gravity.

The influence of the linear density of the weft yarn on the absorption rate in the woven fabrics needs further investigation.

**Figure 10:**
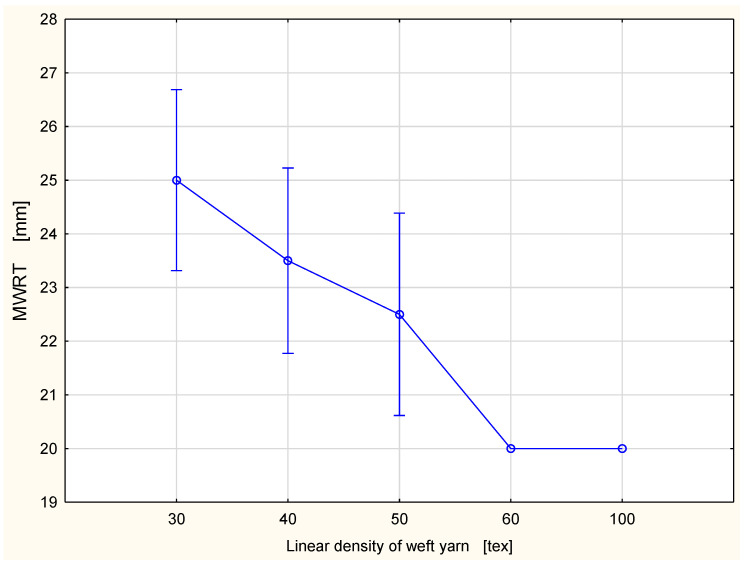
Maximum wetted radius for the top fabric surface vs. linear density of the weft yarn.

**Figure 11:**
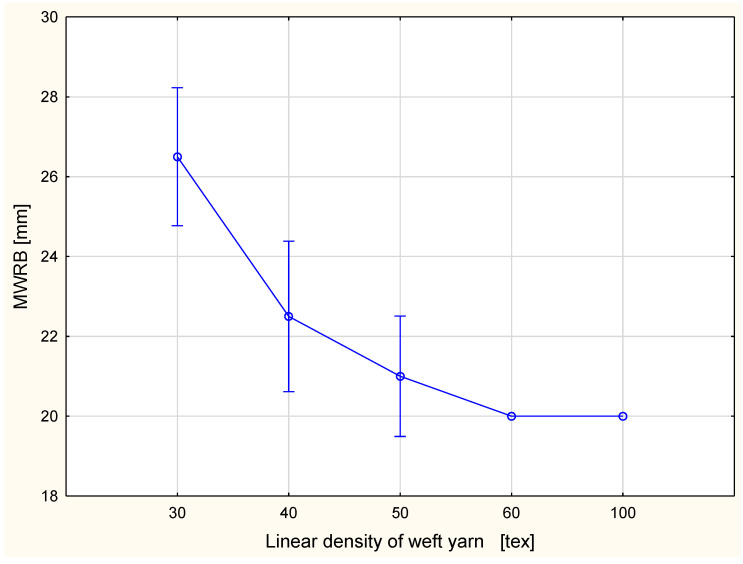
Maximum wetted radius for the bottom fabric surface vs. linear density of the weft yarn.

The biggest maximum wetted radius was found for the fabric variant with the 30 tex weft yarn (Figure 10 and Figure 11). The values were the following: the MWRT was 25.0 mm and the MWRB 26.5 mm. The lowest values of the maximum wetted radius occurred for the fabric variants with the 100 tex and 60 tex weft yarns. For both fabric surfaces, the maximum wetted radius was 20.0 mm. On both surfaces, the maximum wetted radius decreased with the increase of the linear density of the weft yarn. This was according to our expectations. Coarser weft yarn means a larger share of fibrous material in the fabric structure. As was mentioned, the investigated fabrics were made of cotton, which is hydrophilic. A larger amount of cotton causes more intensive absorption of liquid and retention of liquid inside the fibers. This is not conducive to the spread of fluid over the surfaces.

It should be mentioned here that the wetted radius does not mean that the trace of moisture on the fabric surface had a round shape. The radius refers to the maximum radius of the sensor ring on which the presence of moisture was recorded.

**Figure 12:**
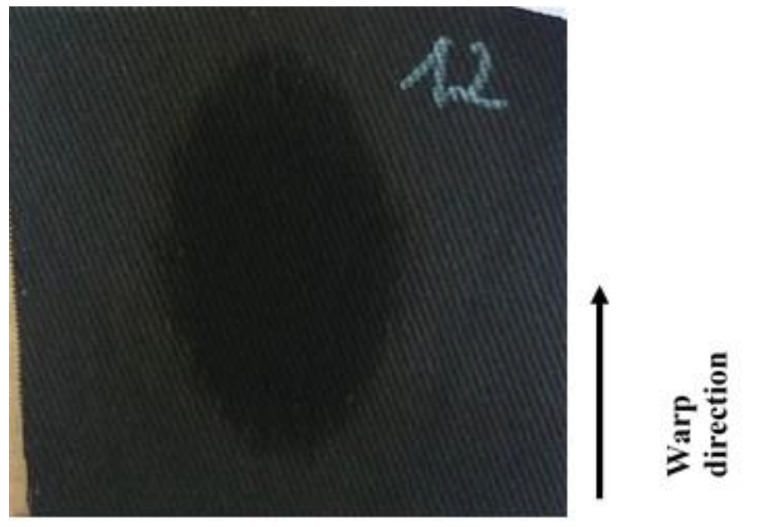
Exemplary picture of the investigated fabric after testing by means of the MMT.

In the case of the investigated fabrics, the shape of trace of moisture on the fabric surface was usually elliptical (Figure 12).

**Figure 13:**
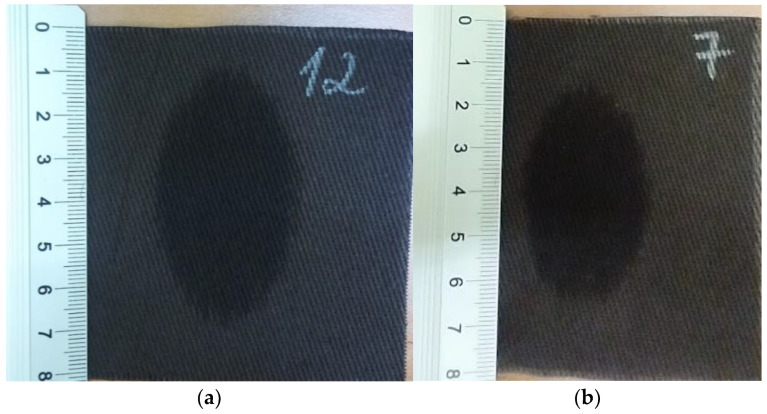
Comparison of liquid trace on variants with (**a**) 30 tex weft yarn and (**b**) 100 tex yarn.

In order to analyze the shape of the trace of spread liquid, the picture of each measured sample was taken directly after the test. This allowed us to assess the influence of the weft yarn on the shape of the liquid trace. In the same way, it was possible to analyze the spreading of the liquid in the warp and weft directions. The pictures below show a comparison of two variants: the 30 tex weft yarn (Figure 13a) and the 100 tex weft yarn (Figure 13b). It was found that the increase of the linear density of the weft yarn caused a decrease of the longer diameter of the ellipse, i.e., in the warp direction.

For each fabric variant, 10 repetitions of measurement were performed. After each test, a picture was taken. Due to this fact, we have 10 pictures of the liquid trace on the fabric surface for each fabric variant. The pictures were analyzed to determine the average diameters in both directions for each variant. In the presented pictures (Figure 13), the difference between diameters in the warp direction of both compared variants is ca. 1 cm.

The accumulative one-way transport index, R, is a measure of the difference between the areas of the liquid moisture content curves of the bottom and the top surfaces of a specimen with respect to time [33]. A fabric with good accumulative one-way transport from the inner fabric side to the outer side (high value of the parameter) offers good sweat management to the wearer. This is due to the fact that with a high accumulative one-way transport index, the fabric keeps the skin of the wearer dry due to the transport of the perspiration towards the outer side of the fabric, which is away from the skin. The positive and high values of the R parameter show that liquid sweat can be transferred from the human skin to the outer surface easily and quickly [35].

**Figure 14:**
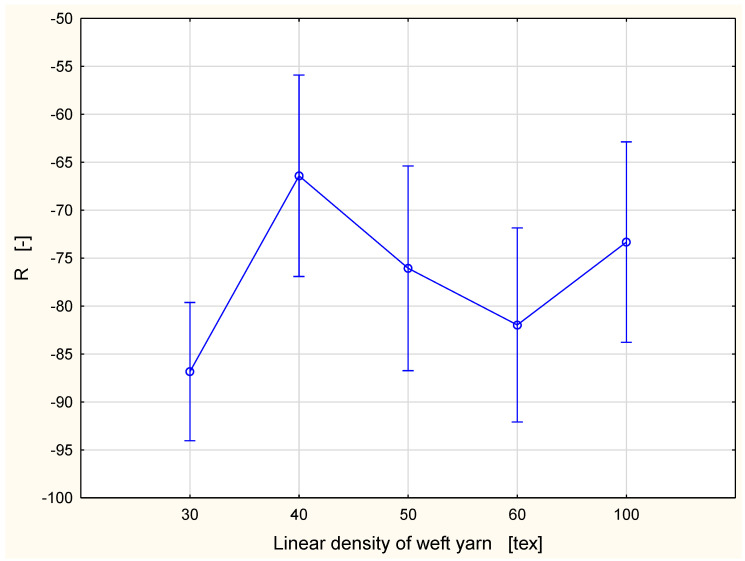
Accumulative one-way transport index vs. linear density of the weft yarn.

For each variant, the value of the R parameter was negative (Figure 14). This means that the fabrics did not transport the liquid from the top surface to the bottom surface well. This was due to the fact that the fabrics accumulated the liquid inside their structure due to the hydrophilicity of the cotton fibers. Additionally, in the case of the R parameter, no clear tendency was observed in relationship between the R value and the linear density of the weft yarn. The highest value was observed for the fabric variant with the 40 tex weft yarn (−66.41), the lowest for the variant with the 30 tex weft yarn (−86.83). Regardless of the absolute value of the R parameter, due to its negative value, all variants of the investigated fabrics can be assessed as not good from the point of view of the liquid moisture transport from the inner to the outer surface of the fabric.

**Figure 15:**
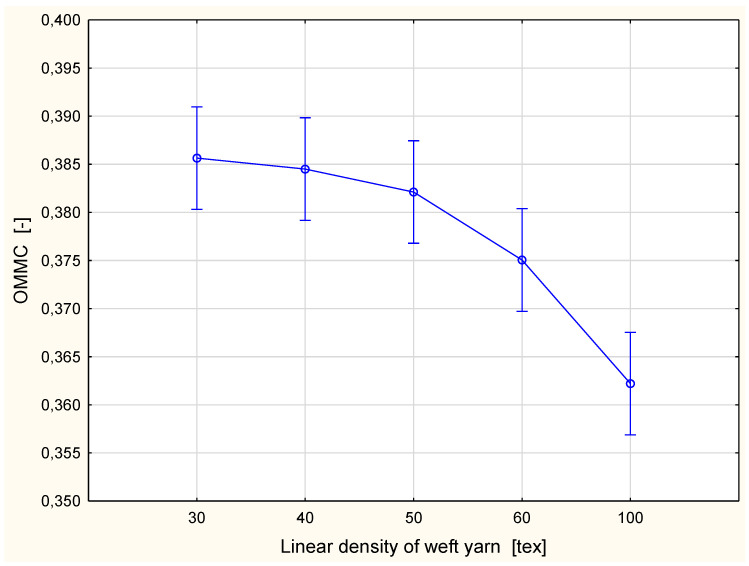
Overall moisture management capacity vs. linear density of the weft yarn.

The OMMC (overall moisture management capacity) was calculated using the formula presented in the AATCC Test Method 195-2017 [34]. The parameter was based on the absorption rate for the bottom surface, the spreading speed for the bottom surface, and the one-way transport capability. The weights of abovementioned parameters were established based on human perception studies. The value of the OMMC parameter can be in the range 0–1. The higher the value of the OMMC parameter is, the better the ability of fabrics to manage liquid moisture. In the case of the investigated cotton fabrics, their ability to manage liquid moisture decreased with the increase of the linear density of the weft yarn (Figure 15). The best result was achieved for the fabric with the 30 tex weft yarn, the worst for the fabric with the 100 tex weft yarn. The drop in the OMMC parameter value was not too great in the range of the weft yarn linear density from 30 tex to 50 tex. Further thickening of the weft yarn caused a more clear decrease in the OMMC value. According to the classification rules proposed by the MMT manufacturer [33], all fabric variants can be assessed as poor from the point of view of liquid moisture management. The differences between the fabric variants investigated are in the range of the poor class.

Due to the fact that the null hypothesis (assuming equality of the mean values of individual parameters for the tested variants of fabrics) was rejected, an analysis of the significance of differences between the individual means was performed using Tukey’s test. The Tukey’s test results for individual parameters are presented in the tables below (Table 5, Table 6, Table 7, Table 8, Table 9, Table 10, Table 11, Table 12, Table 13 and Table 14). In the tables, statistically significant differences are marked in italics and bold.

On the basis of the results of the Tukey’s test, we can compare in pairs the mean values of the parameters from the MMT. In a majority of cases, the differences between particular variants of the investigated fabrics were statistically significant. Notably, the statistically significant differences occurred between the fabric variant with the 100 tex weft yarn and other fabric variants being investigated. This concerned the following parameters: WTT, WTB, SST, SSB, and OMMC. It is easy to explain because the weft yarn used in this variant was significantly thicker than the weft yarns in the rest of the investigated fabric variants. In this fabric variant, the 100 tex weft yarn was more than 3 times thicker than that of the 30 tex weft yarn and twice as thick as that of the 50 tex weft yarn. This means that the fabric variant with the 100 tex weft yarn had 47% greater mass per square meter than the fabric variant with the 30 tex weft yarn. In comparison to other fabric variants this value was: 36% greater than for the fabric with the 40 tex weft yarn, 30% greater than for the fabric variant with the 50 tex weft yarn, and finally 23% greater than for the fabric with the 60 tex weft yarn (Table 1). This reflects the differences in the amount of fibrous material which absorbs liquid moisture easily. Additionally, the coarse weft yarn resulted in higher take-up of the warp yarn (Table 1). This refers to the longer fragments of the warp yarns obliquely running from the top to the bottom surface of the fabric and vice versa. The greater take-up of the warp yarn in the fabric with the 100 tex weft yarn in comparison to the take-up of the warp in the fabrics with thinner weft yarns also explains the phenomenon presented in Figure 13: a shorter diameter of the liquid trace along the warp in the fabric with the 100 tex weft yarn (Figure 13b) than that for the fabric with the 30 tex weft yarn (Figure 13a).

In the case of the TAR and the BAR parameters, statistically significant differences occurred between the fabric variant with the 60 tex weft yarn and other variants being investigated. This was already stated in Figure 8 and Figure 9. As was mentioned earlier, the influence of the linear density of the weft yarn on the absorption rate in the woven fabrics needs further investigation. Probably, the lowest values of the TAR and the BAR parameters stated for the fabric variant with the 60 tex weft yarn are the results of an interaction of all described structural factors.

In the case of the R parameter (accumulative one-way transport capability), a statistically significant difference between means was stated only between the variant pairs with 30 tex weft yarn and 40 tex weft yarn (Table 13). Probably, the rest of the differences between means were assessed as insignificant statistically due to the large dispersion of the results for this parameter (Table 3).

A similar situation was found for the OMMC parameter (Table 14). Statistically significant differences were found only between the fabric variant with the 100 tex weft yarn and other variants being compared in pairs. The explanation presented above of the differences between the fabric variant with the 100 tex weft yarn and other compared fabric variants also applies to the OMMC. However, it is difficult to explain the results for the remaining pairs of the investigated fabric variants. The OMMC is a synthetic parameter calculated on the basis of three other parameters: the BAR, SSB, and R. It can be assumed that the interaction of these parameters caused an equalizing of the differences between analyzed pairs of fabrics. This will be the object of further investigations.

It should be mentioned here that for an assessment of the influence of the weft yarn thickness on an ability of fabrics to transport liquid moisture, the fabric variants were manufactured in the twill 3/1S weave. Further investigations [25] showed that the weave plays a significant role in shaping the liquid moisture management ability of woven fabrics. The twill 3/1 weave turned out to be one of the least favorable. Due to this fact, it is possible to improve the performance of cotton woven fabrics from the aspect of their liquid moisture transport and in the same way from the aspect of their liquid sweat transport and evaporation. This can be done by an appropriate selection of weave and type of yarn. The density of warp and weft also is important in shaping the comfort-related properties of woven fabrics. This will be the subject of another publication.

## 4. Conclusions

In the presented work, five variants of cotton fabrics woven from twill 3/1 S weave were the subjects of investigations. Diversification of fabric structure was achieved by using weft yarns of the linear density 30 tex, 40 tex, 50 tex, 60 tex, and 100 tex (Supplementary Materials). The fabrics were measured by means of the Moisture Management Tester MMT M290 by SDL Atlas in order to assess their ability to ensure liquid moisture transport. This feature of fabrics is very important, especially for fabrics designed for clothing. The liquid moisture transport determines the transport and evaporation of sweat produced by the human body and condensed on the human skin.

The obtained results allowed us to analyze the influence of types of weft yarn on values of the parameters characterizing the liquid moisture transport in cotton woven fabrics. The analysis was performed using the one-way ANOVA and the Tukey’s tests. It was stated that the linear density of the weft yarn influences the parameters characterizing the liquid moisture transport in cotton woven fabrics and that the influence is statistically significant at the significance level 0.05.

On the basis of the performed investigations and obtained results the following conclusions can be drawn:

- The type of the weft yarn significantly influences the parameters characterizing the liquid moisture transport in cotton woven fabrics;
- The fabric variant with the 30 tex weft yarn (the thinnest one among the weft yarns in the investigated fabrics) was characterized by the shortest wetting time and the highest spreading speed for both fabric surfaces. This means that the fabric provides a larger area of contact with liquid in the same time than fabric with a longer wetting time;
- The fabric variant with the 30 tex weft yarn also was characterized by the biggest maximum wetted radius for both sides of the fabric. This means that the fabric ensured spreading the liquid on the biggest area to facilitate faster evaporation of liquid sweat;
- On the basis of a majority of parameters from the MMT it can be stated that the lower the linear density of weft yarn is, the better the performance of cotton woven fabrics from the aspect of liquid moisture transport;
- The influence of the linear density of weft yarn on the accumulative one-way transport capability of cotton woven fabrics needs further investigation; currently stated relationships are difficult to explain;
- It is possible to influence the ability of cotton woven fabrics to transport liquid moisture by an appropriate selection of the structural parameters of fabrics.

## Figures and Tables

**Table 1:** The basic structural parameters of the investigated fabrics.

Parameter	Unit	Linear Density of Weft Yarn (tex)				
		30	40	50	60	100
Warp density	cm^−1^	318	320	317	317	317
Weft density	cm^−1^	118	118	116	117	116
Mass per square meter	g m^−2^	198	215	225	238	292
Take-up—warp	%	4.1	4.5	5.0	5.5	7.9
Take-up—weft	%	4.3	3.2	3.6	3.8	3.3
Thickness	mm	0.61	0.65	0.67	0.70	0.78

**Table 2:** The results from the MMT.

Weft Yarn		WTT	WTB	TAR	BAR	MWRT	MWRB
30 tex	Mean	2.84	3.00	65.62	58.83	25.0	26.5
	SD	0.26	0.17	1.56	1.04	2.4	2.4
40 tex	Mean	3.02	3.20	63.63	57.81	23.5	22.5
	SD	0.21	0.13	0.79	0.99	2.4	2.6
50 tex	Mean	3.23	3.26	63.44	57.04	22.5	21.0
	SD	0.13	0.20	1.05	0.95	2.6	2.1
60 tex	Mean	3.19	3.24	61.59	55.02	20.0	20.0
	SD	0.22	0.15	1.23	1.07	0.0	0.0
100 tex	Mean	3.69	3.88	65.13	58.09	20.0	20.0
	SD	0.23	0.27	1.48	1.38	0.0	0.0

**Table 3:** The results from the MMT; continuation.

Weft Yarn		SST	SSB	R	OMMC
30 tex	Mean	5.83	5.83	−86.83	0.38
	SD	0.31	0.20	10.07	0.00
40 tex	Mean	5.13	4.96	−66.41	0.38
	SD	0.34	0.27	14.69	0.00
50 tex	Mean	4.68	4.46	−76.06	0.38
	SD	0.22	0.17	14.93	0.01
60 tex	Mean	4.51	4.37	−81.96	0.38
	SD	0.18	0.23	14.14	0.003
100 tex	Mean	3.80	3.76	−73.33	0.36
	SD	0.15	0.20	14.61	0.016

**Table 4:** The results of the one-way ANOVA.

Parameter	SS_efect_	df_effect_	MS_effect_	SS_error_	df_error_	MS_error_	F	*p*
WT T	3.9560	4	0.9890	2.1055	45	0.0468	21.1371	0.0000
WT B	4.3202	4	1.0800	1.6381	45	0.0364	29.6687	0.0000
TAR	100.6410	4	25.1602	70.7904	45	1.5731	15.9938	0.0000
BAR	84.7286	4	21.1822	54.0948	45	1.2021	17.6209	0.0000
MWRT	193.0000	4	48.2500	165.0000	45	3.6667	13.1591	0.0000
MWRB	295.0000	4	73.7500	155.0000	45	3.4444	21.4113	0.0000
SS T	22.6624	4	5.6656	2.8468	45	0.0633	89.5558	0.0000
SS B	24.0451	4	6.01128	2.0877	45	0.0464	129.5706	0.0000
R	2477.119	4	619.2797	8580.4080	45	190.6757	3.2478	0.0201
OMMC	0.003759	4	0.000940	0.003155	45	0.0001	13.4029	0.0000

Legend: SS—sum of squares, MS_effect_—mean square of effect expressing the between-groups variability, MS_error_—mean square of error expressing the within-group variability, df—degree of freedom, F—variable of F distribution, *p*—significance level.

**Table 5:** The results of the Tukey’s test for the WTT.

Linear Density of Weft Yarn	30 tex	40 tex	50 tex	60 tex	100 tex
30 tex		0.367577	***0.002412***	***0.007315***	***0.000134***
40 tex	0.367577		0.225014	0.419045	***0.000134***
50 tex	***0.002412***	0.225014		0.995060	***0.000333***
60 tex	***0.007315***	0.419045	0.995060		***0.000197***
100 tex	***0.000134***	***0.000134***	***0.000333***	***0.000197***	

**Table 6:** The results of the Tukey’s test for the WTB.

Linear Density of Weft Yarn	30 tex	40 tex	50 tex	60 tex	100 tex
30 tex		0.163752	***0.037799***	0.064023	***0.000134***
40 tex	0.163752		0.964598	0.992215	***0.000134***
50 tex	***0.037799***	0.964598		0.999520	***0.000134***
60 tex	0.064023	0.992215	0.999520		***0.000134***
100 tex	***0.000134***	***0.000134***	***0.000134***	***0.000134***	

**Table 7:** The results of the Tukey’s test for the TAR.

Linear Density of Weft Yarn	30 tex	40 tex	50 tex	60 tex	100 tex
30 tex		***0.008017***	***0.003166***	***0.000134***	0.910537
40 tex	***0.008017***		0.997395	***0.006388***	0.072193
50 tex	***0.003166***	0.997395		***0.015789***	***0.032651***
60 tex	***0.000134***	***0.006388***	***0.015789***		***0.000134***
100 tex	0.910537	0.072193	***0.032651***	***0.000134***	

**Table 8:** The results of the Tukey’s test for the BAR.

Linear Density of Weft Yarn	30 tex	40 tex	50 tex	60 tex	100 tex
30 tex		0.247170	***0.005935***	***0.000134***	0.561270
40 tex	0.247170		0.522028	***0.000140***	0.979080
50 tex	***0.005935***	0.522028		***0.001565***	0.221178
60 tex	***0.000134***	***0.000140***	***0.001565***		***0.000134***
100 tex	0.561270	0.979080	0.221178	***0.000134***	

**Table 9:** The results of the Tukey’s test for the MWRT.

Linear Density of Weft Yarn	30 tex	40 tex	50 tex	60 tex	100 tex
30 tex		0.413968	***0.041456***	***0.000137***	***0.000137***
40 tex	0.413968		0.769452	***0.001707***	***0.001707***
50 tex	***0.041456***	0.769452		***0.041456***	***0.041456***
60 tex	***0.000137***	***0.001707***	***0.041456***		1.000000
100 tex	***0.000137***	***0.001707***	***0.041456***	1.000000	

**Table 10:** The results of the Tukey’s test for the MWRB.

Linear Density of Weft Yarn	30 tex	40 tex	50 tex	60 tex	100 tex
30 tex		***0.000290***	***0.000134***	***0.000134***	***0.000134***
40 tex	***0.000290***		0.382305	***0.032931***	***0.032931***
50 tex	***0.000134***	0.382305		0.748637	0.748637
60 tex	***0.000134***	***0.032931***	0.748637		1.000000
100 tex	***0.000134***	***0.032931***	0.748637	1.000000	

**Table 11:** The results of the Tukey’s test for the SST.

Linear Density of Weft Yarn	30 tex	40 tex	50 tex	60 tex	100 tex
30 tex		***0.000134***	***0.000134***	***0.000134***	***0.000134***
40 tex	***0.000134***		***0.002437***	***0.000157***	***0.000134***
50 tex	***0.000134***	***0.002437***		0.559993	***0.000134***
60 tex	***0.000134***	***0.000157***	0.559993		***0.000134***
100 tex	***0.000134***	***0.000134***	***0.000134***	***0.000134***	

**Table 12:** The results of the Tukey’s test for the SSB.

Linear Density of Weft Yarn	30 tex	40 tex	50 tex	60 tex	100 tex
30 tex		***0.000134***	***0.000134***	***0.000134***	***0.000134***
40 tex	***0.000134***		***0.000174***	***0.000135***	***0.000134***
50 tex	***0.000134***	***0.000174***		0.902112	***0.000134***
60 tex	***0.000134***	***0.000135***	0.902112		***0.000134***
100 tex	***0.000134***	***0.000134***	***0.000134***	***0.000134***	

**Table 13:** The results of the Tukey’s test for the R.

Linear Density of Weft Yarn	30 tex	40 tex	50 tex	60 tex	100 tex
30 tex		***0.015328***	0.417892	0.932825	0.203667
40 tex	***0.015328***		0.529177	0.104588	0.795069
50 tex	0.417892	0.529177		0.872825	0.991930
60 tex	0.932825	0.104588	0.872825		0.632122
100 tex	0.203667	0.795069	0.991930	0.632122	

**Table 14:** The results of the Tukey’s test for the OMMC.

Linear Density of Weft Yarn	30 tex	40 tex	50 tex	60 tex	100 tex
30 tex		0.998130	0.878659	0.051395	***0.000134***
40 tex	0.998130		0.967995	0.102810	***0.000136***
50 tex	0.878659	0.967995		0.338452	***0.000167***
60 tex	0.051395	0.102810	0.338452		***0.011004***
100 tex	***0.000134***	***0.000136***	***0.000167***	***0.011004***	

## Data Availability

The data presented in this study are available on request from the corresponding author.

## Supplementary Materials

The following supporting information can be downloaded at: https://www.mdpi.com/article/10.3390/ma15186489/s1, Results from the MMT M290 are presented in the Tables S1–S5.
