# Supplementary material for: Liquid Moisture Transport in Cotton Woven Fabrics with Different Weft Yarns
Source: Materials (Basel). 2022 Sep 19;15(18):6489. doi: 10.3390/ma15186489 (PMC9504572; doi:10.3390/ma15186489)
Supplement: Supplementary file 1 [file materials-15-06489-s001.zip › materials-1872961-supplementary.pdf]

RESULTS OF FABRICS' MEASUREMENT USING THE MMT M290

Table S1. Results of fabrics' measurement using the MMT M290 for the woven fabric with the 100 tex weft yarn.

Repetition	Top Wetting Time	Bottom Wetting Time	Top Absorption Rate	Bottom Absorption Rate	Top Max Wetted Radius	Bottom Max Wetted Radius	Top Spreading Speed	Bottom Spreading Speed	Accumulative One-way transport index (%)	OMMC
	[s]	[s]	[%/s]	[%/s]	[mm]	[mm]	[mm/sec]	[mm/sec]	[-]	[-]
BR 7-1	3.556	3.837	65.6045	58.6362	20	20	3.9934	4.1385	-76.4073	0.385
BR 7-2	3.837	4.493	66.2492	60.347	20	20	3.9052	3.8135	-79.1167	0.374
BR 7-3	3.744	3.744	63.8628	56.3581	20	20	3.7755	3.6355	-51.8593	0.348
BR 7-4	4.212	4.119	67.5196	58.4256	20	20	3.6155	3.4517	-69.0119	0.339
BR 7-5	3.464	3.932	62.882	57.9553	20	20	3.9939	3.7474	-70.0305	0.362
BR 7-6	3.369	3.557	63.4167	55.9496	20	20	3.6045	3.5093	-73.2789	0.337
BR 7-7	3.557	3.65	64.1814	57.6621	20	20	3.7172	3.7956	-108.2979	0.365
BR 7-8	3.744	3.744	65.6753	57.2176	20	20	3.843	3.8404	-68.0552	0.368
BR 7-9	3.744	3.931	65.6691	58.5517	20	20	3.6709	3.7371	-61.8223	0.363
BR 7-10	3.651	3.744	66.2878	59.7745	20	20	3.9142	3.9047	-75.4263	0.38

Table S2. Results of fabrics' measurement using the MMT M290 for the woven fabric with the 60 tex weft yarn.

Repetition	Top	Bottom	Top	Bottom	Top	Bottom	Top	Bottom	Accumulative One-way transport index (%)	OMMC
	Wetting Time	Wetting Time	Absorption Rate	Absorption Rate	Max Wetted Radius	Max Wetted Radius	Spreading Speed	Spreading Speed		
	[s]	[s]	[%/s]	[%/s]	[mm]	[mm]	[mm/sec]	[mm/sec]	[-]	[-]
BR 8-1	3.089	2.996	62.5908	55.5181	20	20	4.7029	4.705	-84.1718	0.376
BR 8-2	3.557	3.182	62.8143	53.9805	20	20	4.4663	4.1868	-83.8542	0.372
BR 8-3	3.183	3.37	62.5641	56.575	20	20	4.668	4.3909	-97.8971	0.379
BR 8-4	2.902	3.276	62.301	56.3407	20	20	4.7683	4.7409	-71.4952	0.379
BR 8-5	3.276	3.089	62.8926	54.6036	20	20	4.3046	4.2194	-108.1773	0.374
BR 8-6	3.369	3.276	61.2444	55.6948	20	20	4.4684	4.2486	-57.2665	0.377
BR 8-7	2.901	3.182	60.2022	54.0153	20	20	4.615	4.4331	-87.4954	0.372
BR 8-8	2.995	3.182	59.7701	53.3941	20	20	4.5775	4.5535	-78.389	0.371
BR 8-9	3.369	3.556	59.9599	55.51	20	20	4.3063	4.1719	-74.2884	0.376
BR 8-10	3.276	3.276	61.6106	54.5554	20	20	4.2483	4.0948	-76.6069	0.374

Table S3. Results of fabrics' measurement using the MMT M290 for the woven fabric with the 50 tex weft yarn.

Repetition	Top	Bottom	Top	Bottom	Top	Bottom	Top	Bottom	Accumulative One-way transport index (%)	OMMC
	Wetting Time	Wetting Time	Absorption Rate	Absorption Rate	Max Wetted Radius	Max Wetted Radius	Spreading Speed	Spreading Speed		
	[s]	[s]	[%/s]	[%/s]	[mm]	[mm]	[mm/sec]	[mm/sec]	[-]	[-]
BR 9-1	3.182	3.276	62.4974	57.0064	20	20	4.535	4.4053	-73.6227	0.381
BR 9-2	3.182	3.276	63.4935	57.5354	20	20	4.5046	4.454	-36.9277	0.397
BR 9-3	3.276	3.182	63.988	57.2604	20	20	4.5845	4.5974	-85.5781	0.381
BR 9-4	3.37	3.464	64.0398	57.3242	25	20	4.7824	4.3547	-72.9212	0.382
BR 9-5	3.463	3.463	65.5972	58.4374	25	25	4.9388	4.6625	-81.523	0.385
BR 9-6	3.089	3.463	62.8772	58.4286	25	20	4.8771	4.4147	-75.4813	0.385
BR 9-7	3.37	2.995	64.2579	55.8351	25	20	4.7635	4.3824	-84.6211	0.377
BR 9-8	3.089	2.901	62.0607	55.782	25	25	5.0007	4.7703	-91.3344	0.377
BR 9-9	3.089	3.182	62.7055	56.4041	20	20	4.413	4.2288	-81.4838	0.379
BR 9-10	3.183	3.37	62.917	56.3657	20	20	4.4274	4.3244	-77.0636	0.379

Table S4. Results of fabrics' measurement using the MMT M290 for the woven fabric with the 40 tex weft yarn.

Repetition	Top Wetting Time [s]	Bottom Wetting Time [s]	Top Absorption Rate [%/s]	Bottom Absorption Rate [%/s]	Top Max Wetted Radius [mm]	Bottom Max Wetted Radius [mm]	Top Spreading Speed [mm/sec]	Bottom Spreading Speed [mm/sec]	Accumulative One-way transport index (%) [-]	OMMC [-]
BR 11-1	2.995	3.182	63.0848	58.1283	25	25	5.1011	5.1884	-63.7195	0.384
BR 11-2	3.089	3.463	64.3871	59.7731	20	20	4.9591	5.1589	-43.6535	0.395
BR 11-3	3.182	3.089	64.2515	58.0324	25	25	5.3288	5.1153	-41.0109	0.393
BR 11-4	3.182	3.276	63.8529	57.9144	25	20	5.2069	4.6028	-71.5999	0.383
BR 11-5	2.715	2.996	64.2552	57.8059	25	20	5.6268	5.0391	-78.3892	0.383
BR 11-6	3.276	3.276	64.0911	57.7323	20	25	4.553	4.843	-57.2108	0.383
BR 11-7	2.901	3.276	62.6445	58.5847	20	25	4.7868	5.2282	-71.205	0.385
BR 11-8	2.901	3.182	63.4137	56.7715	25	20	5.5703	4.9134	-81.3153	0.38
BR 11-9	2.714	3.089	62.093	56.1664	25	25	5.2712	5.1409	-78.8948	0.378
BR 11-10	3.276	3.183	64.2112	57.1833	25	20	4.8794	4.4229	-77.1238	0.381

Table S5. Results of fabrics' measurement using the MMT M290 for the woven fabric with the 30 tex weft yarn.

Repetition	Top	Bottom	Top	Bottom	Top	Bottom	Top	Bottom	Accumulative One-way transport index (%)	OMMC
	Wetting Time	Wetting Time	Absorption Rate	Absorption Rate	Max Wetted Radius	Max Wetted Radius	Spreading Speed	Spreading Speed		
	[s]	[s]	[%/s]	[%/s]	[mm]	[mm]	[mm/sec]	[mm/sec]	[-]	[-]
BR 12-1	2.621	2.902	62.278	56.2794	25	25	5.9089	5.8035	-106.6793	0.379
BR 12-2	3.183	3.37	67.9426	60.1095	20	25	5.2147	5.6878	-86.5794	0.389
BR 12-3	3.089	2.996	67.2631	59.1214	25	25	5.7326	6.1471	-78.0841	0.386
BR 12-4	3.183	3.183	66.4424	58.6782	25	25	5.581	5.6137	-84.6901	0.385
BR 12-5	2.621	3.089	64.3919	58.9187	25	30	5.7001	5.6586	-79.2829	0.386
BR 12-6	2.901	2.995	65.4818	58.6601	25	30	5.9279	6.0218	-76.4352	0.385
BR 12-7	2.808	2.808	65.1712	58.2727	25	25	5.8227	5.7065	-81.8325	0.384
BR 12-8	2.996	2.996	66.097	59.5591	25	25	5.9164	5.6627	-80.6133	0.388
BR 12-9	2.527	2.808	65.3563	59.0529	30	30	6.3166	6.0876	-97.609	0.386
BR 12-10	2.528	2.902	65.7379	59.6323	25	25	6.2184	5.9429	-96.5028	0.388
